# Cystotomy for Bladder Tumours of Doubtful Malignancy

**Published:** 1895-03

**Authors:** J. Paul Bush

**Affiliations:** Surgeon to the Bristol Royal Infirmary


### CYSTOTOMY FOR BLADDER TUMOURS OF DOUBTFUL MALIGNANCY.

BY

J. Paul Bush, M.R.C.S. Eng.,

*Surgeon to the Bristol Royal Infirmary.*

---

The surgeon of the present day has a most difficult task in deciding whether a tumour of the bladder is a malignant or an innocent growth, and the following case will show how impossible it is to decide this question even when the bladder has been incised and the tumour removed.

W. H., aged 41, came under my care on July 23rd, 1894: there was no family history of any of the hereditary diseases; the patient had had no previous illness; for about a year and a half he had noticed a frequent desire to pass water, and for a little over a year he had noticed that a drop or two of blood-stained urine often appeared at the meatus after difficult micturition; this was accompanied by some slight pain in the penis, lasting only a few minutes. The frequency of the passage of blood gradually increased, and the pain steadily became worse up to July 15th, when the bladder was sounded; this caused very free hemorrhage, and since that date there has been always a large amount of blood passed, mixed with the urine, and always some free bleeding through the urethra after micturition. On July 23rd there was marked difficulty in forcing the urine out of the bladder, and this could be done only in very small quantities at frequent intervals.

On examining the abdomen, the bladder could be felt as a hard rounded mass, reaching about one and a half inches above the pubes, and the finger in the rectum made out what felt like a hard nodular mass involving the whole of the base of the bladder: a sound was with difficulty introduced into the bladder, when it appeared evident that the cavity was almost completely filled with a growth; no stone or phosphatic deposit was detected.

The inguinal glands were enlarged on both sides.

On July 26th, with the patient under ether, I was able to inject about 4 oz. of warm boracic lotion into the bladder; I then performed supra-pubic cystotomy, making a free incision into the bladder; the finger in the bladder could feel a mass of solid, easily broken-down material, adherent to the whole of the anterior wall as well as to the base. The growth was removed freely with a Barker's flushing scoop, leaving what felt like a hard and thickened ulcerating surface involving the whole of the front and lower walls of the bladder; a large india-rubber tube was fixed in the bladder by a suture, and the upper edges of the abdominal wound were brought together with a single stitch.

There was excessive hemorrhage during the whole of the time the operation lasted, and no view of the mucous membrane of the bladder could be obtained, although the opening was a free one.

For the first twenty-four hours there was continuous bleeding from both wound and urethra; in thirty-six hours this was rapidly decreasing in quantity; six days after the operation there was only a trace of blood in the urine, which was still coming freely through the tube above the pubes; seven days later, a smaller tube was introduced into the bladder—about half the urine was at this time being passed *per urethram;* all bleeding had entirely ceased.

Three weeks after the operation the drainage-tube was removed, and shortly after this the opening in the bladder closed.

Patient went home apparently cured on August 31st, when he was passing normal urine about six times during the twenty-four hours.

The portions of growth removed, when heaped together, about equalled in size the half of the man's fist; microscopic examination showed it to be a fibro-papilloma.

Feb. 23rd, 1895. It is now seven months since the operation: the man is in perfect health, and has passed normal urine without pain or difficulty ever since going home; on examining the bladder with a sound, nothing abnormal can be made out, and the examination causes no blood to appear in the urine; with the finger in the rectum, no thickening can be felt at the base of the bladder.

This was a case that appeared to be malignant both before and during the operation; in fact, when the patient was sent back to bed it was much to be feared that the growth had not been entirely removed, as the thickened walls in front and below were left in what seemed to be a ragged ulcerating condition.

Surgeons have until quite recently been most reluctant to attempt to remove a growth from the bladder that was thought to be malignant; but even in those cases of sarcoma and carcinoma involving the bladder only, I have noticed a very marked improvement in the cases in which free curetting has been practised. The supra-pubic operation of cystotomy is now performed with such comparative safety, that the great relief of pain and the diminished hemorrhage that so frequently result, fully justify the operation in even apparently hopeless cases. Moreover, there is at present a great difference of opinion concerning the malignancy of bladder tumours; this is clearly shown by the fact that one authority states that of thirty consecutive cases that came under his notice no less than twenty-six were innocent; while in another consecutive set of twelve cases occurring in hospital, ten were malignant!

I am of opinion that if nearly all cases of tumour of the bladder were operated upon, a vast amount of pain and distress would be saved and a far larger number of cases would be cured.

